# Effect of bilingualism on visual tracking attention and resistance to distraction

**DOI:** 10.1038/s41598-020-71185-6

**Published:** 2020-08-31

**Authors:** Ana Janic, Patrick Cavanagh, Josée Rivest

**Affiliations:** 1 Department of Psychology, Glendon College, York University, 2275 Bayview Avenue, Toronto, ON M4N 3M6 Canada; 2 Centre for Vision Research, York University, Toronto, ON Canada; 3 Department of Psychological and Brain Sciences, Dartmouth College, Hanover, NH USA; 4 Present Address: Institute of Medical Sciences, University of Toronto, Toronto, ON Canada

**Keywords:** Human behaviour, Attention, Visual system

## Abstract

Speaking more than one language has been associated with enhanced cognitive capacities. Here we evaluated whether bilingual individuals have advantages in visual tracking attention. Adult bilingual (n = 35) and monolingual (n = 35) participants were tested in the Multiple Object Tracking task (MOT). In one condition, the MOT was performed by itself establishing the baseline performance of each group, and in the other condition, it was performed while participants counted backward out loud in their mother tongue. At baseline, the average speed tracking threshold of bilinguals was not better than that of the monolinguals. Importantly, for bilinguals, counting backward decreased their threshold by only 15%, but, for monolinguals, it decreased it three times as much. This result suggests that bilingualism confers advantages to visual tracking attention when dual tasking is required, extending the evidence that bilingualism affords cognitive benefits beyond verbal communication.

## Introduction

Communicating in several languages is often a necessity in today’s globalized world; it represents a sophisticated exercise involving selecting one language, inhibiting others, and often switching from one to another. In the present study, we ask whether engaging in this demanding skill affects visual tracking attention and its resistance to distraction. We compare the performance of bilingual individuals to that of monolinguals on a classic test of visual attention, the multiple object tracking task (MOT), and examine how their performance is affected by distraction.

Constant monitoring is required to operate adequately in two or more languages and it has been argued that these processing demands lead to an executive processing advantage in bilinguals^1–5^ (we use the term “bilinguals” to refer to individuals who know two or more languages). Consistent with this claim, bilinguals show a different organization in the frontal areas (executive control networks) than monolinguals^5–7^, and the onset of behavioral symptoms of neurodegenerative disorders are delayed for bilinguals compared to monolinguals^8–11^.

Advantages of attention have been associated with these anatomical and neurological differences. Across many studies, adults bilinguals are either better or no different at monitoring attention than monolinguals, certainly they are never worse^1,2,12–16^. In particular, adult bilinguals often show an advantage in visual attention tasks such as the Flanker and Simon tasks^2,4^ that have conflicting or facilitating cues and require rapid decisions. Moreover, bilinguals are quicker and more accurate at locating a target among distractors and are less influenced by conflicting information, leading Frisen, Latman, Calvo, and Bialystok^17^ and Hernández, Costa, and Humphreys^18^ to propose that bilinguals have superior visual attention skills particularly when attentional demand is high. In these studies, the attention tasks typically involve conflicting cues and rely on cognitive factors like response readiness and decision making. Indeed, these tasks call on exactly those executive functions which have been postulated to be superior in bilinguals.

Here we aim at using a well-established visual attention task, the MOT task, that differs in important ways from previously studied tasks and that has never been used in research on bilingualism. In the MOT, several items move randomly on the display screen and participants must attend to and track a specified set (the targets) while ignoring the rest (the distractors). This task is different in that it requires continuous and dynamic attention—items change their location over time and thus, attention must be deployed continuously in order to keep the targets separate from the distractors. Moreover, from trial to trial, the procedure is the same. There is no variation in cues or response demands and so no differences in stimulus conflicts and decision criteria. We first establish and compare the baseline performance of adult monolinguals and bilinguals in this visual attention task. We then increase the demands on executive control by adding a separate distracting task and assess how both groups respond to this dual task.

Visual attention has often been operationalised using MOT measures. The best known measure of performance for the MOT task is the number of targets that can be tracked with, say, 75% accuracy, typically about four^19^. However, it is well established that the number of targets that can be tracked decreases as the speed of the items increases (e.g.^20–22^). Thus, an alternative measure of performance for the MOT task is the speed at which a fixed number of targets (e.g. three) can be tracked at a certain accuracy level (see^23–30^). This is the measure we use here.

Performance on the MOT has been demonstrated to rely on visual attention (e.g.^31^), attentional selection (e.g.^32^) and working memory (e.g.^33–35^). MOT performance has been shown to be a valid measure of visual attentional function in several studies (review^24^). For example, individuals with expertise in attention demanding tasks like radar operators, video game players, and elite athletes (e.g.^29,35–38^) have better performance and faster learning rates in MOT. There is also strong evidence that MOT shares central attentional resources with other tasks, both visual and non-visual. Tracking performance drops when participants run a second, concurrent task, for example, visual search, or auditory tone discrimination^34,39–41^. Overall, the MOT task shows a strong dependence on visual attention and a sensitivity to interference in dual task situations. These two properties led us to choose the MOT to determine whether bilingual advantages extend to visual attention and to evaluate their resilience to distraction.

Bilinguals may have acquired executive processing advantages in linguistic contexts because of the processing demands for monitoring and switching between languages^1–3,5,42^. However, bilinguals have also demonstrated superior abilities in managing dual tasks outside of linguistic contexts. For example, Söman et al.^43^ compared the verbal memory of bilinguals to that of monolinguals performing a card sorting task while encoding and retrieving a list of words. They showed that bilinguals’ verbal memory is less hindered than that of monolinguals by the dual task. Nevertheless, advantages for bilinguals have not been found in all tasks involving executive functions (see recent reviews and meta analyses^44–48^). Discrepancies across studies may be due to different methodological and sampling approaches, such as different ways of operationalizing executive functions and bilingualism.

Advantages in executive functions may evolve from any extensive experience at regulating multiple codes. For example, musicians are another group like bilinguals who must often regulate the use of two codes, linguistic and musical, and indeed, they too show advantages in executive functions. For example, Poudrier and Repp^49^ showed that musicians are able to track two simultaneous rhythms better than non-musicians. Chweiri, Manoochehri, and Rivest^50^ showed that musicians perform better on visual search tasks while being distracted by sounds.

Here we evaluate how the basic performance at a continuous visual tracking attentional task (MOT) is affected by engaging in a simple separate and continuous counting task, comparing the cost of this dual task between bilinguals and monolinguals. If bilinguals’ MOT results are more resistant to this dual verbal task, it would be evidence that they have enhanced executive control that confers advantages even in the visual domain.

Using the MOT task, we evaluate visual tracking performance by the speed of the moving disks needed to reach a threshold tracking accuracy. The participants are bilingual and monolingual adults of equivalent age, gender and educational background. The bilingual participants have been functioning in two or more languages continuously over a minimum of the past eight years either at home, school and/or work. Monolingual participants are English-speaking adults who have only ever been functioning in English at home, school and/or work.

We take the performance levels of the two groups as a baseline for evaluating the effect of our distraction condition. The MOT task has not yet been tested with bilinguals, and unlike the other visual attention tasks that have been used (e.g. visual search, Simon, Flanker tasks), it requires continuous monitoring and does not involve cue-guided decisions. Consequently, we have no clear reasons to favor bilinguals over monolinguals or vice versa in the baseline task. However, because of their superiority in dual task contexts, we do predict that bilinguals’ performance will be less impacted by a distracting, second task than that of monolinguals. We chose a non-visual, continuous, and low load dual task. Participants continuously count backwards out loud by ones (e.g., 178, 177, 176,…) while simultaneously performing the MOT. We then evaluate the extent to which the distracting task affects the MOT relative to the no distraction baseline.

## Methods

### Participants

Participants were undergraduate students from York University, Glendon College, Toronto, Ontario, Canada. They were all recruited from courses in Psychology. Thirty-five bilingual individuals (25 females) between the ages of 18 and 40 years old (*M* = 20.43, *SD* = 3.06) participated. In order to be included in the study as “bilinguals”, participants had to indicate that they fit the following written description: *“A bilingual individual is someone who has been consistently speaking more than one language for the last eight years in a home, school or work environment.”* Out of the 35 bilinguals who endorsed this description, 33 identified English as their first language; 20 were fluent in two languages, 12 in three, and three in four or more. Age-, gender-, and education-matched, 35 monolingual English-speakers (22 females) between the ages of 18 and 40 years old (*M* = 20.03, *SD* = 2.74) participated. All monolinguals reported using English only in their life, and none of them has ever been functioning in any other languages at home, school, or work. As Canadian students, participants took courses of French or English as a second language in their schooling years.

### Procedure and material

The MOT task was accessed through an open-source website program: https://lab.tellab.org/show/paradigm/mot/5a019f3971a894c407e1430e and ran on a 13″ 2017 MacBook Pro. The display screen was viewed at a distance of about 51 cm. The refresh rate was 60 Hz.

**Figure 1:**
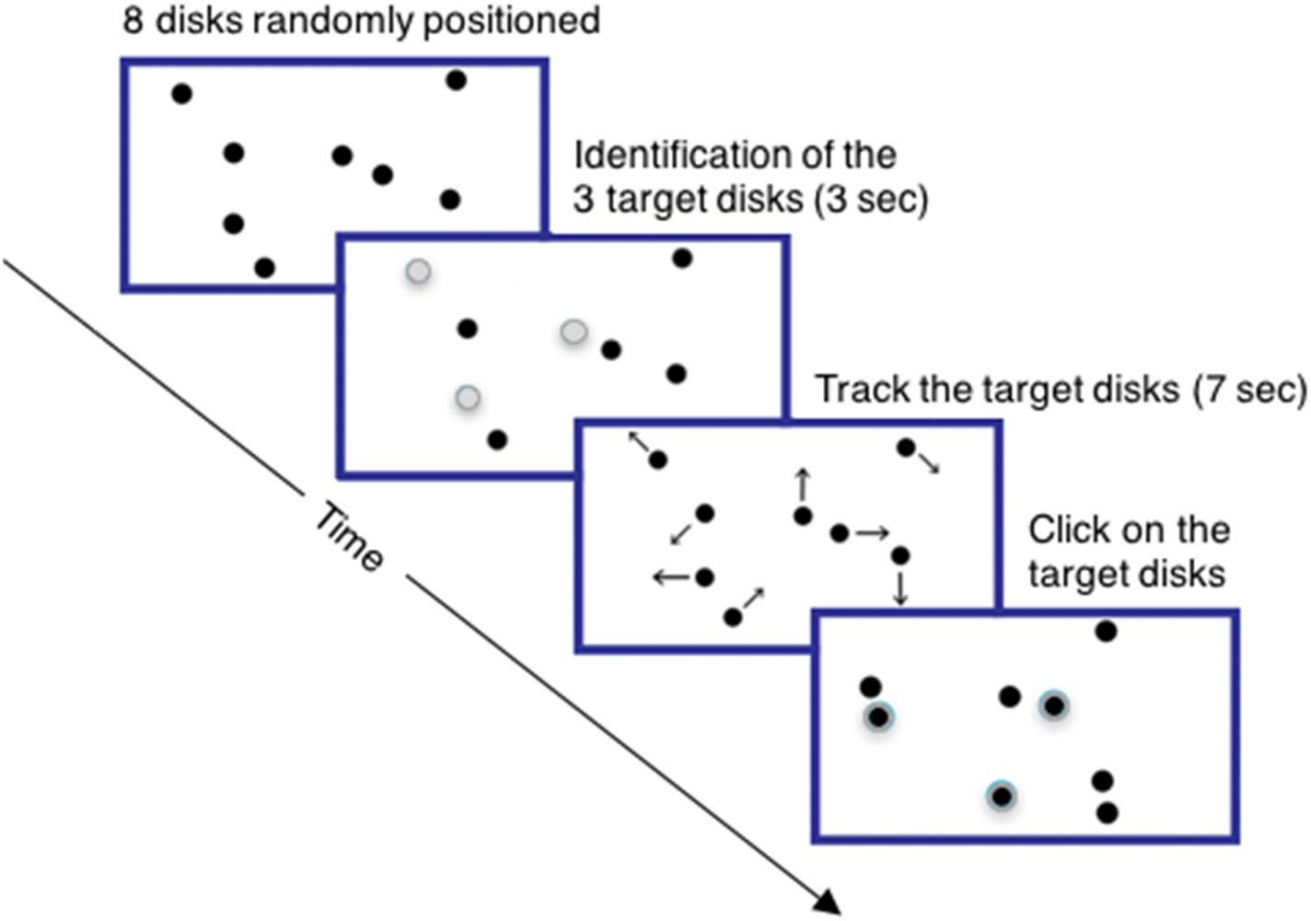
Schematic illustration of one MOT trial.

On each trial, eight black disks (1.1° radius) moved around within a white box (18.2° × 13.7°) and collided with each other and the box boundaries randomly, creating a display of non-overlapping bouncing disks. At the start of a trial, three randomly chosen bouncing disks expanded and contracted for 300 ms; they had to be tracked as the target disks. All disks became identical again for 7 s during which participants had to track the target disks with attention. As in most MOT studies, participants were allowed to move their eyes freely, as eye movements alone can only keep track of one of three targets^51^. After 7 s, the disks stopped moving and participants used the trackpad to click on the three balls they believed to be the targets. Feedback was provided for each ball selected (Fig. 1 schematically illustrates one trial.)

There were five testing blocks—the speed of the disks increased for each sequential block, from 3.58, 4.86, 6.14, 7.42, to 8.68°/s. There were 10 trials per speed, and the number of disks correctly selected was recorded for each trial. The average proportion correct for each speed was plotted and a Probit function was fitted to these data. The speed at which a participant had 67% correct answers was determined from the Probit fit. This 67% value is the speed threshold and represents a performance near mid-way between a chance level performance (picking correctly 3 disks out of 8 by chance: 37.5%) and a perfect performance (100%). A faster speed threshold indicates better visual tracking abilities as participants can keep a tracking accuracy at 67% correct while the discs move faster.

Each participant performed this MOT task twice (in an order that alternated across participants); once as described above and once while doing a distracting task. This secondary task had to engage attention continuously for the full duration of each tracking trial, had to be non-visual and easy for both groups (monolinguals and bilinguals), and had to avoid additional distraction from any external cueing. These dual-task criteria were met by having participants count backwards by 1 s, out loud, at a steady speed of about one digit per second while performing the MOT task as described above. Just before the beginning of each MOT trial, a random number between 150 and 500 was given to participants. They immediately had to continuously count backward out loud in their mother tongue until they selected the target disks. The experimenter monitored the counting in order to make sure that all numbers were accurate, voiced out loud and that the pace was regular (at about 1 number per second). Any participant who could not comply with the counting task requirements was to be eliminated. No one was removed: monolinguals and bilinguals performed the counting task without difficulty.

All participants gave informed consent and the study was approved by the Glendon Psychology, Delegated Ethics Research Review Committee, York University. As such, all methods of study were carried out in accordance with the declaration of Helsinki guidelines and regulations.

## Results

**Figure 2:**
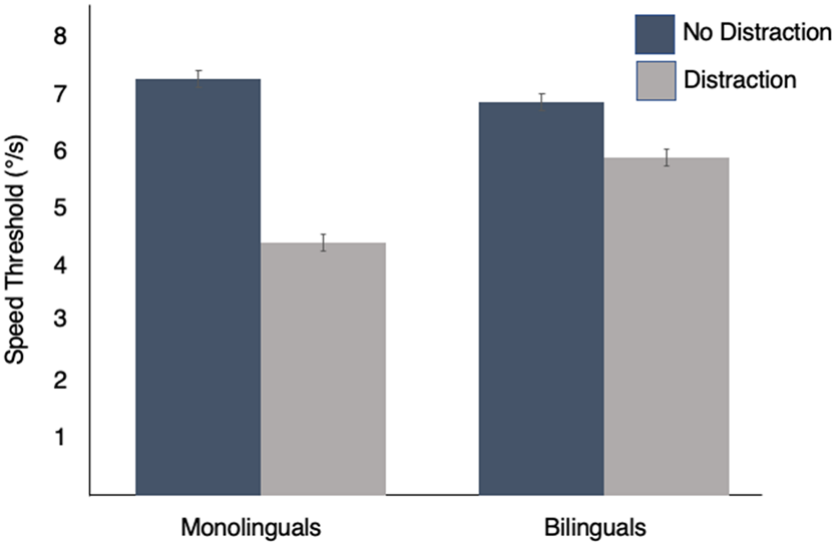
Average speed threshold of bilinguals vs. monolinguals in the baseline and in the distraction task. Each error bar represents the 95% Confidence Interval.

For each participant, the percent correct responses (total number of correctly identified targets over 10 trials, for a maximum of 30 targets × 100) was plotted for each of the five speeds assessed. A Probit function was fitted to these data, and the speed (degrees/second) at which each participant obtained 67% correct (correctly identified a total of 20 targets over 30 in 10 trials) was determined. This speed threshold (in °/s) represented the dependent measure for each participant. The average speed threshold was determined for the MOT task in the baseline (no distraction) and in the distraction conditions for both groups (see Fig. 2).

A 2 × 2 mixed-design ANOVA with Language Ability (monolingual vs. bilingual) as a between-subject variable and MOT task (without and with distraction) as a within-subject variable was performed on the speed thresholds. The main effects of Language Ability [F (1, 68) = 9.53, p < 0.003, η^2^ = 0.06, P_r_ = 0.86] and MOT task [F (1, 68) = 438.19, p < 0.0001, η^2^ = 0.75, P_r_ = 1.00] were both significant. As well, there was a significant interaction between Language Ability and MOT task, [F (1, 68) = 30.57, p < 0.0001, η^2^ = 0.18, P_r_ = 1.00]. The difference in the average speed thresholds of bilinguals and monolinguals when performing the MOT task by itself (baseline) [bilinguals’ threshold: 6.80 (*SD* = 0.84°/s) vs. monolinguals’: 7.19 (*SD* = 0.92°/s)] was not significant and its effect size was quite small [two-tailed t (68) = 1.88, p = 0.06, η^2^ = 0.05, P_r_ = 0.45]. Though this difference approached significance, if it existed, it was slightly in favor of monolinguals: they were able to perform the visual tracking task as accurately as bilinguals when the balls moved slightly faster (by 0.93°/s).

Although the monolinguals may have a small advantage for the baseline MOT task in isolation, the outcome was quite different when the distracting task, counting backwards, was added. In that case, the monolinguals’ threshold dropped significantly below that of bilinguals [*M* = 4.36, *SD* = 0.62°/s vs. *M* = 5.84, *SD* = 0.89°/s for monolingual and bilinguals, respectively; two-tailed t (68) = 8.06, p < 0.0001, η^2^ = 0.50, P_r_ = 1.00]. For bilinguals, counting backward decreased their threshold by an average of 0.96°/s (*SD* = 0.73), but for monolinguals, it decreased three times as much, by an average of 2.83°/s (*SD* = 0.79) [two-tailed t (68) = 10.32, p < 0.0001, η^2^ = 0.61, P_r_ = 1.00].

## Discussion

The results show that the performance of bilinguals on the isolated tracking task was not better than that of monolinguals. However, when dual tasking, bilinguals only had a slight decrease in performance whereas the monolinguals showed a dramatic loss in performance.

Without distraction, bilinguals showed no advantage in the visual attention task; if anything, they had a small disadvantage. These similar baseline performances between bilinguals and monolinguals demonstrate that both groups had relatively similar performance and engagement in the task. Other studies that did show visual attention advantages in adult bilinguals involved multiple levels of attentional processing such as shifting, engaging and disengaging attention (e.g.^2,4,17,18,52^). Why would bilinguals show an advantage for these tasks but not for the MOT task (without distraction)? MOT requires only one type of spatial attention –continuous tracking of targets of interest, whereas the other tasks called on several aspects of attentional control. We can speculate that the advantages of executive functions that come with bilingualism may be specific to only some of the attentional processes or only to higher level attentional control. Whatever the case, if our result is replicated with other bilingual samples where MOT is directly compared to other attention tasks, these results could reveal important distinctions among the processing of various visual attention skills.

The absence of an MOT advantage for bilinguals contrasts with the MOT advantage that is found in individuals with expertise in visual attentional skills such as radar operators, and video game players (e.g.^35–38^). It is possible that these differences are due to the sensory domain of the expertise: Bilinguals have expertise in the verbal domain whereas the other individuals who showed better MOT results were expert in the visual domain.

Consistent with previous reports, MOT performance was reduced with the dual task (e.g.^33,34,40,41^). MOT’s susceptibility to dual tasking has lead researchers to conclude that it shares central attentional resources with other tasks, both visual and non-visual. Nevertheless, the resilience of bilinguals to this interference was remarkable; it may be related to their developed executive processing, and is consistent with the idea that bilingual brains may develop extra cognitive reserves (e.g.^53–56^). Visual tracking may show advantages from bilingualism only when the task is rendered more demanding, which, in turn, requires the engagement of the frontal executive system (as suggested by Bialystok and colleagues). Bilingual advantages found in other visual attention tasks involving conflict resolution and quick decision making^57–60^ have also been attributed to executive function advantages related to activation of frontal areas.

Our results do not allow us to describe what exact mechanism is behind the bilinguals’ resilience to distraction. On one hand, the more efficient executive control of the two concurrent tasks (MOT and counting) may divert fewer attentional resources away from the MOT task. On the other hand, the two concurrent tasks may call on independent attention resources (e.g.^23^) so that the resources required for counting are kept more separate from those related to visual tracking.

It is interesting to compare the anatomy involved in the MOT task to our results. MOT execution has been related to bilateral activation in the parietal lobe, to an attention control center in the frontal lobe (particularly the Frontal Eye Field) and to the MT complex^61^. Culham, Cavanagh, and Kanwisher^62^ showed that the MOT activates two systems—one related to the task independently of the attentional load (the FEF and parietal area 7), and one related to attentional load (the parietal and frontal areas). More direct evidence of the importance of the parietal areas comes from patients with parietal damage who are impaired at the MOT task in the contralesional field^63,64^. Based on our results, we could speculate that while the efficiency of the attentional control network underlying MOT performance per se is not improved by bilingualism, it is more resilient to extra cognitive load. This resilience may be attributed to the frontal areas involved in the MOT task, which show advantages due to bilingualism (Bialystok, Hilchey and colleagues). It is reasonable to speculate that the frontal areas are critically related to the bilingual advantages in the MOT performance while dual tasking.

Bilinguals seem to benefit from extra efficiency in the system related to controlling multiple attentional loads during dual tasks. While performing similarly to monolinguals on the basic task, adding a demand to their attention generated only moderate interference with the resources required for visual tracking compared to that seen for monolinguals. This could be a new manifestation of the resilience of the executive frontal system resulting from improvements in multitasking that develop with bilingualism. Using the MOT with other bilingual samples is warranted.
